# Supplementary material for: Dynalign II: common secondary structure prediction for RNA homologs with domain insertions
Source: Nucleic Acids Res. 2014 Nov 21;42(22):13939–48. doi: 10.1093/nar/gku1172 (PMC4267632; doi:10.1093/nar/gku1172)
Supplement: SUPPLEMENTARY DATA [file supp_gku1172_nar-02021-z-2014-File012.zip › manual/Overview/Components.html]

RNAstructure Installation and Overview: Software Package Components


|  |  |  |
| --- | --- | --- |
|  | RNAstructure Installation and Overview Software Package Components | - Contents - Index |
| Single Sequence Methods Feature \| Text Interface Program \| JAVA/Windows GUI Menu Item \| Class Library and Function Name; Free energy minimization and generation of all suboptimal structures (References: Duan et al. 2006, Wuchty et al. 1999) \| AllSub \| Generate All Suboptimal RNA Structures \| RNA::GenerateAllSuboptimalStructures; Drawing secondary structure diagrams \| draw \| Draw \| RNA::DetermineDrawingCoordinates; Efn2 (Energy function 2) (Reference: Mathews, Sabina et al. 1999) \| efn2 \| Efn2 RNA \| RNA::CalculateFreeEnergy; Free energy minimization structure prediction (Reference: Mathews et al. 2004) \| Fold \| Fold RNA Single Strand \| RNA::FoldSingleStrand; Maximum expected accuracy structure prediction (Reference: Lu et al. 2009) \| MaxExpect \| MaxExpect: Predict RNA MEA Structure \| RNA::MaximumExpectedAccuracy; NAPSS (Reference: Hart et al. 2008) \| NAPSS \| N/A \| N/A; Partition function (Reference: Mathews 2004) \| partition \| Partition Function RNA \| RNA::PartitionFunction; Partition function, implemented in parallel for CUDA GPUs \| partition-cuda \| N/A \| N/A; Prediction of structures with pairs above specified pairing probability threshold (Reference: Mathews 2004) \| ProbablePair \| Output Probable Structures \| RNA::PredictProbablePairs; Prediction of secondary structures including pseudoknots (Reference: Bellaousov and Mathews 2010) \| ProbKnot \| ProbKnot: Predict RNA MEA Structure Including Pseudoknots \| RNA::ProbKnot; Remove Pseudoknots Reference: (Smit et al. 2008) \| RemovePseudoknots \| Break Pseudoknots \| RNA::BreakPseudoknot; Predict structures that may contains pseudoknots, restrained by SHAPE mapping data. \| ShapeKnots \| N/A \| N/A; Stochastic sampling of structures (Reference: Ding and Lawrence, 2003) \| stochastic \| Stochastic RNA Sampling \| RNA::Stochastic Multiple Sequence Methods (Bimolecular or Common Folding) Feature \| Text Interface Program \| JAVA/Windows GUI Menu Item \| Class Library and Function Name; Bimolecular structure prediction with intramolecular pairs \| bifold \| Fold RNA Bimolecular \| HybridRNA::FoldBimolecular; Bimolecular partition function (no intramolecular pairs) \| bipartition \| Partition Function RNA Bimolecular \| HybridRNA::PartitionFunctionBimolecular; Bimolecular structure prediction without intramolecular pairs \| DuplexFold \| N/A \| HybridRNA::FoldDuplex; Dynalign (References: Harmanci et al. 2007, Uzilov et al 2006, Mathews and Turner 2002) \| dynalign \| RNA Dynalign \| Dynalign\_object::Dynalign; Dynalign II \| dynalign\_ii \| N/A \| Dynalign\_object::Dynalign (Built by defining DYNALIGN\_II at precompile time); Multilign (Reference: Xu and Mathews, 2011) \| multilign \| RNA Multilign \| Multilign\_object::ProgressiveMultilign; OligoScreen Reference: Mateeva et al. 2003) \| oligoscreen \| OligoScreen \| Oligowalk\_object::OligoScreen; OligoWalk (References: Lu and Mathews, 2008, Lu and Mathews, 2007, Mathews, Burkard et al. 1999) \| OligoWalk \| OligoWalk \| Oligowalk\_object::OligoWalk; PARTS (References: Harmanci et al. 2009, Harmanci et al. 2008) \| PARTS \| N/A \| N/A; TurboFold References: Harmanci et al. 2011) \| TurboFold \| RNA TurboFold \| TurboFold::fold Utilities Feature \| Text Interface Program \| JAVA/Windows GUI Menu Item \| Class Library and Function Name; Circular structure comparison \| CircleCompare \| N/A \| N/A; CT file to dot bracket file conversion \| ct2dot \| N/A \| N/A; Dot bracket file to CT file conversion \| dot2ct \| N/A \| N/A; Create a dot plot from a Dynalign save file \| DynalignDotPlot \| Dot Plot Dynalign \| N/A; Create a dot plot from a folding energy save file \| EnergyPlot \| Dot Plot \| N/A; Ensemble energy calculation \| EnsembleEnergy \| N/A \| N/A; Create a dot plot from a partition function save file \| ProbabilityPlot \| Dot Plot Partition Function \| N/A; Refold from a previously folded sequence. \| refold \| Refold From Save File \| RNA::ReFoldSingleStrand; Scoring comparison of two structures (Reference: Mathews, Sabina et al. 1999) \| scorer \| N/A \| N/A --- References 1. Harmanci, A.O., Sharma, G. and Mathews, D.H. (2011). TurboFold: Iterative probabilistic estimation of secondary structures for multiple RNA sequences. *BMC Bioinformatics*. 12:108. 2. Xu, Z., and Mathews, D.H. (2011). Multilign: An algorithm to predict secondary structures conserved in multiple RNA sequences. *Bioinformatics*, 27:626-632. 3. Bellaousov, S., and Mathews, D. H. (2010. ProbKnot: fast prediction of RNA secondary structure including pseudoknots. *RNA*. 16:1870-1880. 4. Harmanci, A.O., Sharma, G. and Mathews, D.H. (2009). Stochastic sampling of the RNA structural alignment space. *Nucleic Acids Res.*, 37:4063-4075. 5. Lu, Z.J., Gloor, J.W. and Mathews, D.H. (2009). Improved RNA Secondary Structure Prediction by Maximizing Expected Pair Accuracy. *RNA*, 15:1805-1813. 6. Harmanci, A.O., Sharma, G. and Mathews, D.H. (2008). PARTS: Probabilistic Alignment for RNA joinT Secondary structure prediction. *Nucleic Acids Res.*, 36:2406-2417. 7. Hart, J.M., Kennedy, S.D., Mathews, D.H. and Turner, D.H. (2008). NMR-assisted prediction of RNA secondary structure: identification of a probable pseudoknot in the coding region of an R2 retrotransposon. *J. Am. Chem. Soc.*, 130:10233-10239. 8. Lu, Z.J. and Mathews, D.H. (2008). Fundamental Differences in the Equilibrium Considerations for siRNA and Antisense Oligodeoxynucleotide Design. *Nucleic Acids Res.*, 36:3738-3745. 9. Smit, S., Rother, K., Heringa, J. and Knight, R. (2008). From knotted to nested RNA structures: a variety of computational methods for pseudoknot removal. *RNA*, 14:410-416. 10. Harmanci, A.O., Sharma, G. and Mathews, D.H. (2007). Efficient Pairwise RNA Structure Prediction Using Probabilistic Alignment Constraints in Dynalign. *BMC Bioinformatics*, 8:130. 11. Lu, Z.J. and Mathews, D.H. (2007). Efficient siRNA Selection Using Hybridization Thermodynamics. *Nucleic Acids Res.*, 36:640-647. 12. Duan, S., Mathews, D.H. and Turner, D.H. (2006). Interpreting oligonucleotide microarray data to determine RNA secondary structure: application to the 3' end of Bombyx mori R2 RNA. *Biochemistry*, 45:9819-9832. 13. Uzilov, A.V., Keegan, J.M. and Mathews, D.H. (2006). Detection of non-coding RNAs on the basis of predicted secondary structure formation free energy change. *BMC Bioinformatics*, 7:173. 14. Mathews, D.H. (2004). Using an RNA secondary structure partition function to determine confidence in base pairs predicted by free energy minimization. *RNA*, 10:1178-1190. 15. Mathews, D.H., Disney, M.D., Childs, J.L., Schroeder, S.J., Zuker, M. and Turner, D.H. (2004). Incorporating chemical modification constraints into a dynamic programming algorithm for prediction of RNA secondary structure. *Proc. Natl. Acad. Sci. USA*, 101:7287-7292. 16. Ding, Y. and Lawrence, C.E. (2003). A statistical sampling algorithm for RNA secondary structure prediction. *Nucleic Acids Res.*, 31:7280-7301. 17. Matveeva, O.V., Mathews, D.H., Tsodikov, A.D., Shabalina, S.A., Gesteland, R.F., Atkins, J.F. and Freier, S.M. (2003). Thermodynamic criteria for high hit rate antisense oligonucleotide design. *Nucleic Acids Res.*, 31:4989-4994. 18. Mathews, D.H. and Turner, D.H. (2002). Dynalign: An algorithm for finding the secondary structure common to two RNA sequences. *J. Mol. Biol.*, 317:191-203. 19. Mathews, D.H., Burkard, M.E., Freier, S.M., Wyatt, J.R. and Turner, D.H. (1999). Predicting oligonucleotide affinity to nucleic acid targets. *RNA*, 5:1458-1469. 20. Mathews, D.H., Sabina, J., Zuker, M. and Turner, D.H. (1999). Expanded sequence dependence of thermodynamic parameters provides improved prediction of RNA secondary structure. *J. Mol. Biol.*, 288:911-940. 21. Wuchty, S., Fontana, W., Hofacker, I.L. and Schuster, P. (1999). Complete suboptimal folding of RNA and the stability of secondary structures. *Biopolymers*, 49:145-165. | | |
| Visit The Mathews Lab RNAstructure Page for updates and latest information. | | |
